# Evolving Roles of Natural Terpenoids From Traditional Chinese Medicine in the Treatment of Osteoporosis

**DOI:** 10.3389/fendo.2022.901545

**Published:** 2022-05-16

**Authors:** Yue Zhuo, Meng Li, Qiyao Jiang, Hanzhong Ke, Qingchun Liang, Ling-Feng Zeng, Jiansong Fang

**Affiliations:** ^1^ Science and Technology Innovation Center, Guangzhou University of Chinese Medicine, Guangzhou, China; ^2^ Guangdong Provincial Key Laboratory of Research in Structural Birth Defect Disease, Women and Children’s Medical Center, Department of Pediatric Surgery, Guangzhou Institute of Pediatrics, Guangzhou Guangzhou Medical University, Guangzhou, China; ^3^ Department of Cancer Immunology and Virology, Dana-Farber Cancer Institute, Harvard Medical School, Boston, MA, United States; ^4^ The Third Affiliated Hospital of Southern Medical University, Guangzhou, China; ^5^ The 2nd Affiliated Hospital of Guangzhou University of Chinese Medicine, Guangzhou, China

**Keywords:** osteoporosis, traditional Chinese medicine, terpenoids, osteoblast, osteoclast

## Abstract

Osteoporosis (OP) is a systemic metabolic skeletal disease which can lead to reduction in bone mass and increased risk of bone fracture due to the microstructural degradation. Traditional Chinese medicine (TCM) has been applied in the prevention and treatment of osteoporosis for a long time. Terpenoids, a class of natural products that are rich in TCM, have been widely studied for their therapeutic efficacy on bone resorption, osteogenesis, and concomitant inflammation. Terpenoids can be classified in four categories by structures, monoterpenoids, sesquiterpenoids, diterpenoids, and triterpenoids. In this review, we comprehensively summarize all the currently known TCM-derived terpenoids in the treatment of OP. In addition, we discuss the possible mechanistic-of-actions of all four category terpenoids in anti-OP and assess their therapeutic potential for OP treatment.

## Introduction

As a systemic skeletal disease, Osteoporosis (OP) is characterized by increased risk of bone fragility, chronic pain, and even disability, leading to decreased life quality. Especially, OP strongly affects postmenopausal women and elderly population. About 30-50% of women and those who are more than 70 years old suffer from OP-induced fractures throughout their lives (1–3). In health condition, osteoblasts (OBs, bone-forming cells) and osteoclasts (OCs, bone-resorbing cells) form a balance for bone homeostasis. The lack of OB function or over-activated OC status will disturb the balance and induce OP.

In recent years, there has been a growing interest in traditional Chinese medicine (TCM) for the treatment of OP, such as Liu-Wei-Di-Huang Wan (formula), Morindae Officinalis Radix (herb), Longspur epimedium glycoside (natural product) (4). TCM has accumulated extensive experience for thousands of years and owns fewer adverse effects during a long-term usage comparing to some chemically synthesized medicines (5). Chinese herbal medicines usually play their therapeutic roles through a “multi-components, multi-targets, multi-pathway” mode, which is compatible with the multifactorial nature of OP. Plenty of evidence suggest that targeting OCs with TCM is an efficient strategy for the treatment of OP (6–8).

According to the theory of TCM on the pathogenesis and symptoms of OP, the kidney stores essence, turns it into bone marrow, nourishes bones to strengthen the skeleton, and promotes bone growth and repair. Therefore, ‘kidney deficiency’ is regarded as the underlying cause of all skeletal pathologies (9, 10). Many classic and empirical formulas of TCM used to tonify the kidney are clinically applied in OP treatment, TCMs like Liu Wei Di Huang Wan, Qing E Wan, Jiawei Yanghe Decoction, Er Zhi Wan, Qiangji Jianli Yin, Zuo Gui Wan, Rongjin Tablets, and You Gui Wan showed excellent anti-OP efficacy through reinforcing the kidney (8). Modern pharmacological studies have shown that these classic formulas significantly inhibited OC formation and bone resorption, and promoted bone formation to increase bone mineral density (BMD) (8, 9). Moreover, many individual herbs that make up the formulas of TCM are beneficial for bone formation since they are bone-specific drugs for the treatment of bone fractures and bone loss diseases (11). *Rehmanniae Radix* has been clinically used for more than 3,000 years in Chinese medicine, which has an anti-OP effect through modulating the kidney and liver functions and improving blood circulation (12). Over 140 individual compounds have been isolated from *Rehmanniae Radix*, and iridoid glycosides (a kind of monoterpenoids) are vital for the anti-OP activity of *Rehmanniae Radix* (6).

Terpenoids are structurally diverse and may represent the most diverse source of essential chemotherapeutic drugs. They are isoprene units (C_5_H_8_)n-based nature products and are classified into monoterpenes, sesquiterpenes, diterpenes, triterpenes, and tetraterpenes. To date, more than 40,000 different terpenoids have been obtained in nature (13, 14). Terpenoids are also reported to have anti-inflammatory, anti-cancer, and neuroprotective effects, with beneficial effects on human health. Although the treatment of OP using TCM has a long history and natural terpenoids have been extensively studied for their therapeutic activities against bone resorption (15), less attention has been given to the whole series of terpenoids in the treatment of OP. Therefore, we here summarize anti-OP advances and molecular mechanisms of terpenoids isolated from TCM.

## Natural Terpenoids Against OP

**Figure 1:**
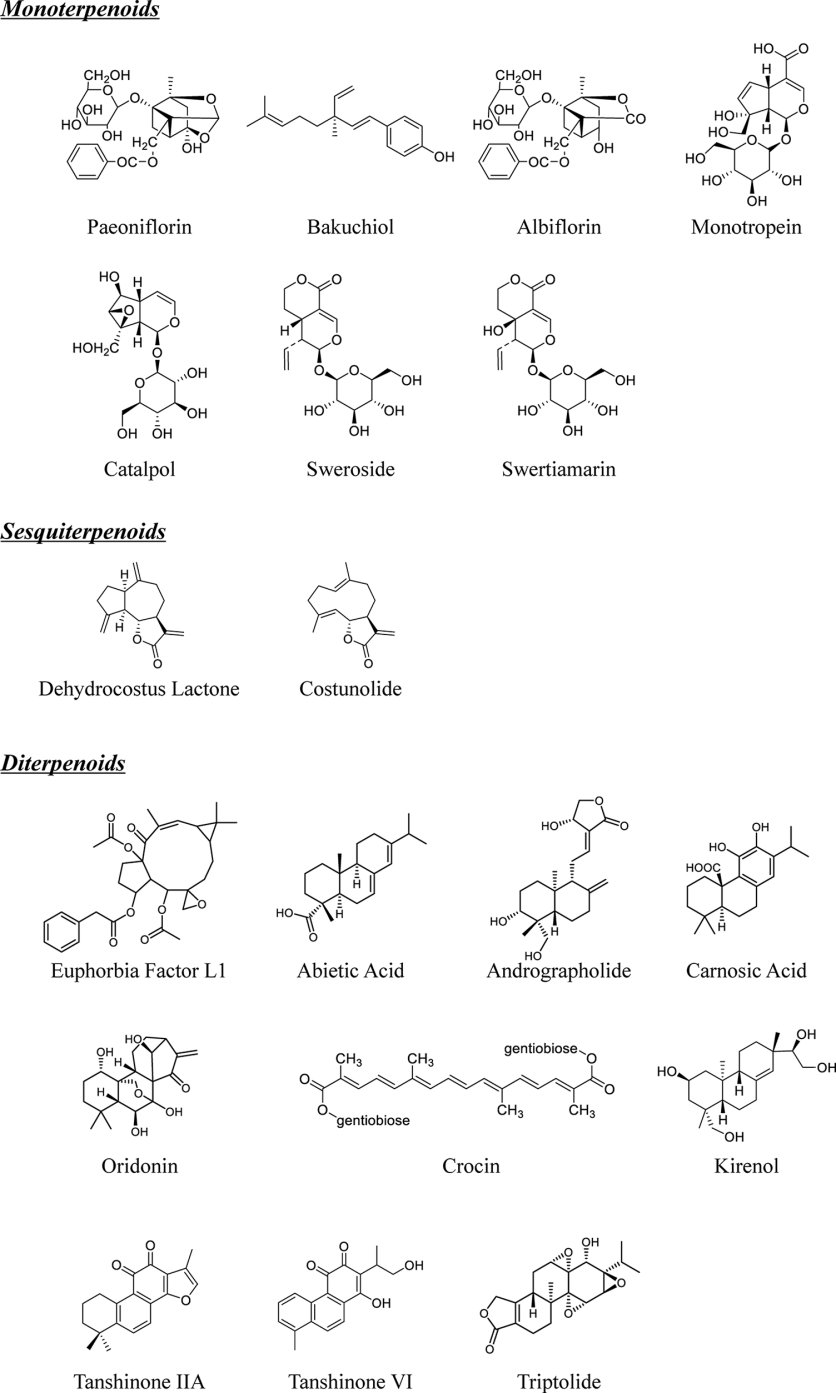
Chemical structures of natural monoterpenoids, sesquiterpenoids and diterpenoids from TCM.

**Figure 2:**
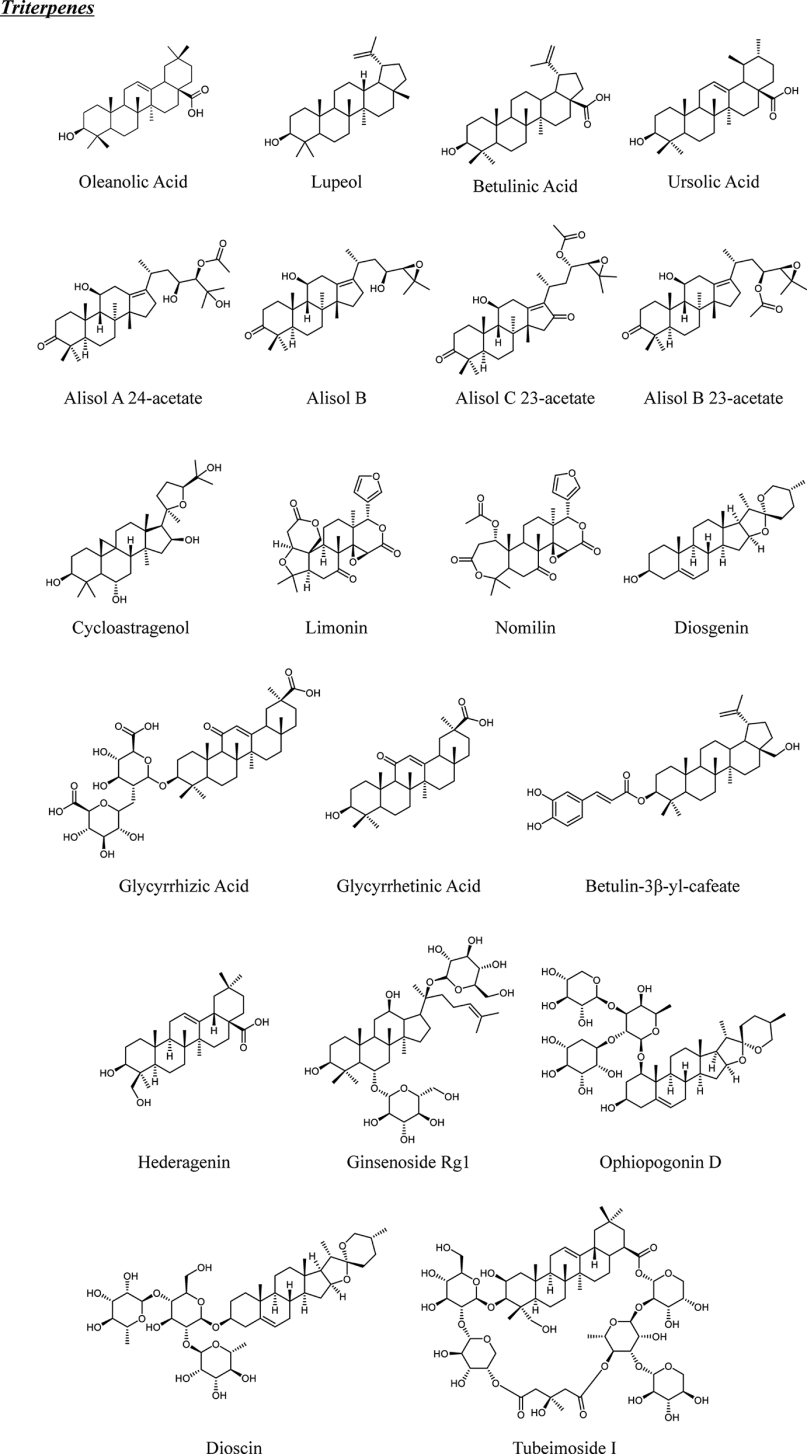
Chemical structures of natural triterpenes from TCM.

**Figure 3:**
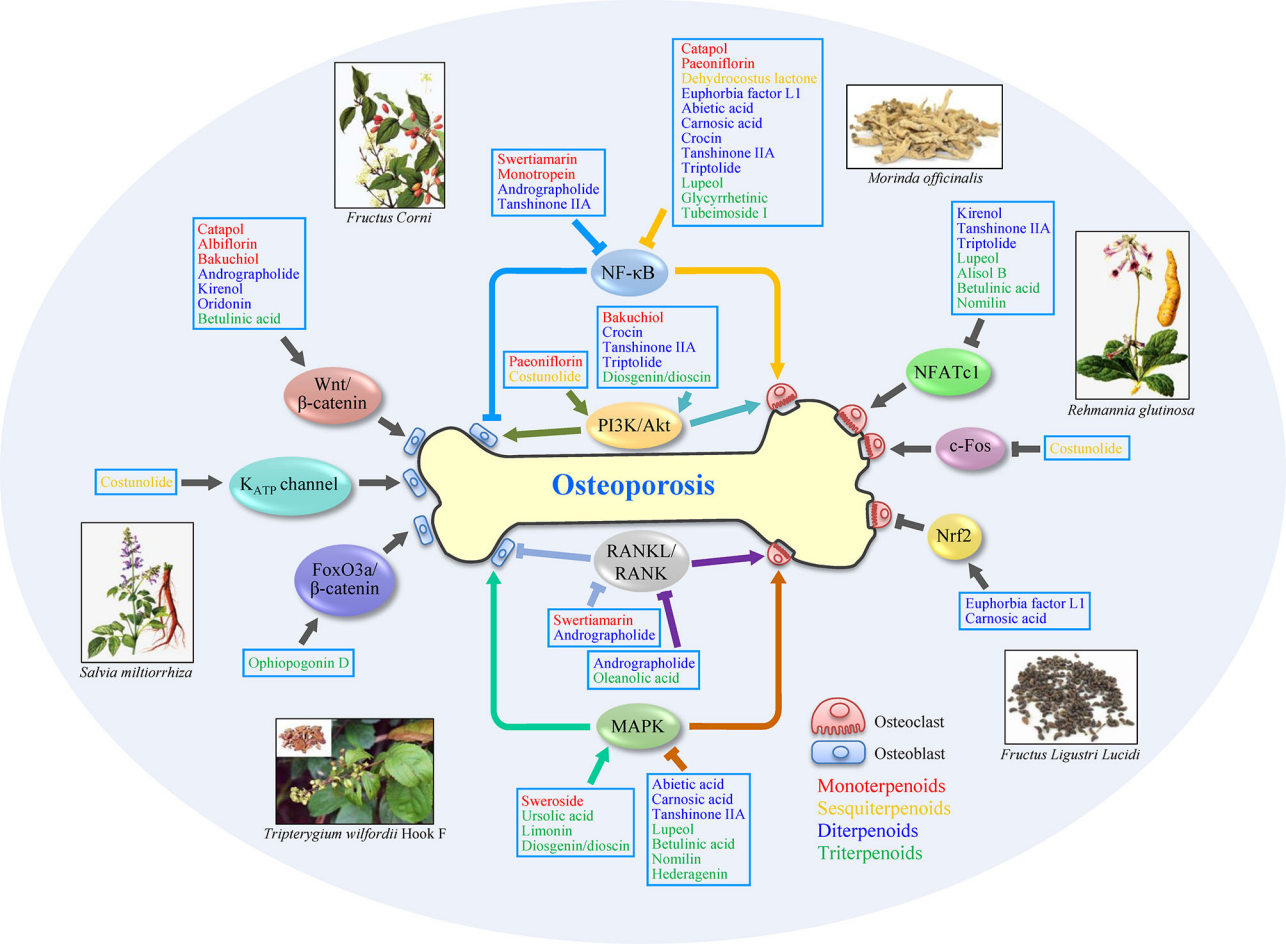
Schematic of anti-osteoporosis mechanisms of terpenoid on osteoblasts and osteoclasts. The activation of MAPK, PI3K/Akt, Wnt/β-catenin signaling pathways and so on, or inhibition of NF-κB and RANKL/RANK signaling pathways, can promote cell proliferation or differentiation in osteoblasts, which benefits osteoporosis treatment. Meanwhile, the inhibition of MAPK, NF-κB, RANKL/RANK, and NFATc1 signaling pathways, or activation of PI3K/Akt and Nrf2 signaling pathways, also exerts potential therapeutic efficacy *via* regulating osteoclasts. Some terpenoids, such as andrographolide and tanshinone IIA, show anti-osteoporosis effect by modifying multi-targets. Arrows (↓) indicate activation of a factor or positive effect on indicated cell type, while inverted T marks (⊥) indicate inhibition or negative effect. Subclass of terpenoids is distinguished with different colors: monoterpenoids (red), sesquiterpenoids (yellow), diterpenoids (blue), and triterpenoids (green).

Terpenoids are classified as monoter-, sesquiter-, diter-, triter-, and tetra-penoids according to different structures (**Figures 1** and **2**). Although few natural terpenoids exhibit genotoxicity or carcinogenicity based on epigenetic mechanism, most are beneficial to humans (15). Natural terpenoids from TCM have been reported to regulate OBs and OCs *via* different signaling pathways (concluded in **Figure 3** and **Table 1**), such as nuclear factor-κB (NF-κB), Wnt/β-catenin, mitogen-activated protein kinases (MAPK), and receptor activator of nuclear factor-κB ligand (RANKL)/receptor activator of nuclear factor-κB (RANK). We will provide a comprehensive review of natural terpenoids from TCM and their potential in OP therapy.

**Table 1:** Summary of studies for the antiosteoporotic effects of natural terpenoids from natural Chinese medicine.

Category	Compound	TCM	Cells/*in vivo* model	Mechanism	Reference
Monoterpenoids	Sweroside	*Cornus officinalis*	Human osteosarcoma cell line (SaOS-2); OVX mice	Induced the mineralization of bone matrix *via* promoting BMP2/CBFA1	(16)
			Human MG-63 cells; Rat OBs	Promoted differentiation and inhibited apoptosis	(17)
			MC3T3-E1 cells	Activated p38 signaling pathway	(18)
			BMSCs; OVX mouse	Hyperactivated the mTORC1/PS6 signaling pathway	(19)
	Swertiamarin	*Enicostema axillare*	Rat fibroblast-like synoviocytes (FLS)	Inhibited caspase 3, TNFa, IL-6, PGE2, COX-2, iNOS, MMPs, p38 MAPKa and modulated RANKL	(20)
			RAW 264.7 macrophage cells	Inhibited NF-κB/IκB and JAK2/STAT3 signaling	(21)
			C57/BL6J BMCs; Sprague Dawley rat neonates OBs; Freund’s Complete Adjuvant induced rat arthritis	Inhibited RANKL/RANK; promoted OPG signaling	(22)
	Monotropein	*Morinda officinalis*	MC3T3-E1 cell line; Female osteoporotic C57/BL6 mice	Blocked NF-κB pathway; Enhanced bone formation and blocked increased secretion of inflammatory cytokines	(23)
			Primary OBs	Prevented Akt/mTOR signaling pathway	(24)
			MC3T3-E1 cells; OVX C57/BL6 mice	Inhibited sRANKL signaling	(25)
	Catalpol	*Rehmannia glutinosa*	BMMs; RAW264.7 cells; C57BL/6 mice	Suppressed NF-κB and AKT signaling pathways	(26)
			BMSCs; Male Sprague-Dawley rats	Activated Wnt/β-catenin pathway.	(27)
			BMSCs; SD female rats	Activated JAK2/STAT3 axis	(28)
			MC3T3-E1 cells; Male ICR mice	Inhibited bone resorption *via* the OPG/RANKL pathway; enhanced bone formation by regulating IGF-1/PI3K/mTOR pathways	(29)
	Paeoniflorin	*Paeonia lactifloral*	MC3T3-E1 cells	Enhanced glyoxalase system and inhibited the glycation	(30)
			Mice BM cells; Mice OC; RAW 264.7 cells; Male DBA/1 mice; Male C57/BL6 mice	Suppressed NF-κB signaling pathway	(31)
			Mouse BMMs; OVX C57BL/6 mice	Inhibited NF-κB signaling pathway	(32)
			Murine osteoblastic MC3T3-E1 cells	Activated PI3K signaling pathway	(33)
	Albiflorin		MC3T3-E1 cells	Suppressed oxidative damage through protecting cytochrome c and cardiolipin	(34)
			MC3T3-E1 cells; Sprague Dawley rats femoral fractures	Activated BMP-2/Smad and Wnt/β-catenin pathway	(35)
	Bakuchiol	*Psoralea corylifolia*	Primary human OBs; OVX rats Sprague-Dawley rats	Up-regulated the Wnt signalling pathway	(36)
			MCF-7 cells; OVX Sprague–Dawley rats	Increased alkaline phosphatase, Ca concentrations, serum E2 concentration and bone mineral density, and decreased the inorganic P level	(37)
			Primary mouse OC precursor cells; Bone marrow cells	Inhibited AKT and AP-1 pathways	(38)
Sesquiterpenoid	Costunolide	*Saussurea lappa*	Murine OB MC3T3-E1 cells	Activated PI3K signaling pathway	(39)
			Mice BMCs	Suppressed RANKL-mediated c-Fos transcriptional activity	(40)
	Dehydrocostus lactone		Mice BMMs, BMSCs, RAW264.7 cells; OVX C57BL/6J mice	Suppressed NF-κB and NFAT signaling pathways	(41)
			Mice BMMs; Male C57BL/6 mice	Modulated NF‐κB signalling pathway	(42)
			RAW264.7 cells, Mice BMMs (C57BL/6 male mice)	Inhibiting NF-κB and AP-1 pathways	(43)
			Mice BMMs; OVX C57BL/6 female mice	Down-regulated the integrin b3, PKC-b, and Atg5 expression	(44)
Diterpenoids	Euphorbia factor L1	*Euphorbia lathyris*	Mouse BMMs; C57BL/6 male mice	Attenuated c-Fos expression and NF-κB activation; activated Nrf2 signaling pathway	(45)
	Abietic acid	*Pimenta racemosa*	RAW 264.7 cell line; Mice BMMs; C57/BL6 male mice	Inhibited NF-KB and MAPK signaling	(46)
	Andrographolide	*Andrographis paniculata*	BMSC; SD rat	Activated wnt/β-catenin signaling pathway	(47)
			Mouse BMMs; RAW 264.7 cells; OVX C57BL/6 mice	Suppressed RANKL signaling pathways	(48)
			MC3T3-E1 cell; OVX Sprague Dawley rats	Up-regulated the OPG/RANKL signaling pathway	(49)
			Mouse BMSCs; OVX Sprague Dawley rats	Inhibited the NF-kB signaling pathway	(50)
			Mouse BMM Cells; C57/BL6 mice	Attenuated NF-κB and ERK/MAPK signalling pathways	(51)
	Carnosic acid	*Salvia officinalis*	RAW 264.7 cells; Mouse BMMs; C57BL/6 male mice	Activated the Nrf2 and suppressed the NF-κB pathways	(52)
			RAW 264.7 cells; Mouse BMMs; Female C57BJ/6L mice	Dual-targeting of sterol regulatory element-binding protein 2 and ERRα	(53)
	Crocin	*Crocus sativus*	RAW264.7 cells	Regulated glyoxalase, oxidative stress, and mitochondrial function	(54)
			Mice BMMs; Murine macrophage cell line; RAW264.7 cells	Suppressed NF- B signaling pathway	(55)
	Kirenol	*Siegesbeckia orientalis*	Mouse BMMs; OVX C57BL/6 mice	Inhibited Cav-1, NFATc1 and the related NF-κB/MAPKs/c-Fos signaling pathways	(56)
			MC3T3-E1 cells	Activated the BMP and Wnt/β-catenin signaling pathways	(57)
	Tanshinone IIA	*Salvia miltiorrhiza*	Human embryonic kidney (HEK) 293 cells; C57BL/6 mice	Inhibited renin activity	(58)
			Mice osteoblasts; Female Wnt1^sw/sw^ mice	Up-regulated the NF-κB signaling pathway	(55)
	Tanshinone VI		Mice BMMCs; RAW-264.7 cells; C57BL/6 mice	Suppresed the NF-κB, PI3-kinase/Akt, and MAPK pathways, as well as the transcription factor NFATc1	(59)
			Mice bone marrow cells; Male ICR mice.	Inhibited the expression of c-Fos and NFATc1	(60)
			OCs	Inhibited RANKL expression and NFkB induction	(61)
	Triptolide	*Tripterygium wilfordii*	Male Sprague-Dawley rats	Down-regulated RANKL and up-regulated OPG	(62)
			RAW 264.7 (mouse macrophage)	Inhibited NF-kB activation, inhibited IkBa kinase activation, IkBa phosphorylation, and IkBa degradation	(63)
			RAW 264.7 cells; Mice BMMCs; Female C57BL/6 mice	Inhibited PI3K-AKT-NFATc1 pathway	(64)
	Oridonin	*Rabdosia rubescens*	Mouse BMMs; MC3T3-E1 cells; Female C57BL/J6 mice(OVX mice)	Inhibited IκB phosphorylation and Ifrd1 degradation	(65)
			Mouse BMSCs; Mouse BMMs; SD rats	Activated Wnt/β-catenin signaling pathway, down-regulated RANKL and up-regulated OPG expression *in vitro*	(66)
Triterpenoids	Lupeol	*Bombax ciba*	UMR-106 cell; Female Wistar albino rats	Inhibited MAPK, NF- κB, NFATc1, and c-Fos	(67)
	Alisol B 24-acetate	*Alismatis rhizoma*	Mast cells; Balb/c mice, ICR mice	Released Syk-mediated down-stream signals including PLC, ERK, and p38 MAPK, NF-κB, cPLA_2_, COX-2, and Ca^2+^,	(68)
	Alisol B		OBs; Bone marrow cells; ddY mice; C57BL/6J (B6) (wild-type) mice	Inhibit NFATc1 and c-Fos signaling pathway	(69)
	Alisol C 23-acetate		Calvaria osteoblastic cell; OCs; OVX rat	Inhibited RANKL-induced osteoclast differentiation and function	(70)
	Alisol A 24-acetate		Mouse BMCs; BMMs	Downregulated NFATc1	(71)
	Oleanolic acid	*Ligustri lucidi*	Cell Counting Kit-8 (CCK-8); Mouse BMMs; OVX C57BL/6 mice	Inhibited the expression of NFATc1 and suppressed the expression of MMP9, Ctsk, TRAP and Car2	(72)
			RAW264.7 cells	Inhibited RANKL-induced osteoclastogenesis *via* ERα/miR-503/RANK signaling pathway	(73)
	Ursolic acid	*Ligustri lucidi*	Mouse osteoblastic MC3T3-E1 subclone 4 cells	Activated MAP kinases and NF-κB signaling pathway	(74)
	Glycyrrhizic acid	*Glycyrrhiza glabra*	Male Sprague–Dawley rats	Inhibited the 11β-hydroxysteroid dehydrogenase type 1 enzyme (11β-HSD1)	(7)
			RAW264.7 cell; Mouse BMMs; C57BL/6/Bkl mice (OVX mouse)	Suppressed NF-κB, ERK, and JNK pathway	(75)
			RAW264.7 cells; Mouse BMMs;OVX C57BL/6J mice	Inactivated NF-κB signaling.	(76)
			Male CSF1r-eGFP-KI mice and their wild type strain C57BL/6	Diminished the size of inflammatory osteolysis *via* the number of CXCR4+OCPs and TRAP+osteoclasts, decreased the senescence-associated secretory phenotype markers, and elevated the senescence-protective markers	(77)
	Glycyrrhetinic Acid		Mouse BMMs; RAW264.7 cells; OVX C57BL/6 female mice	Inhibited NF-κB and MAPK signaling pathways.	(78)
	Ginsenoside Rg1	*Panax ginseng*	Human dental pulp stem cells (hDPSCs); BMSCs; OCs	Promoted the proliferation and differentiation of DPSCs into odontoblast-like cells by promoted the expression of anti-osteoporosis related genes	(79)
	Betulinic Acid	*Betula pubescens*	Mouse BMMs; Female C57BL/6 mice; OVX mice	Inhibited MAPK and NFATc1 signaling pathways	(80)
			MC3T3-E1 OBs	Activated BMP/Smad/Runx2 and β-catenin signal pathways	(81)
	Limonin	*Evodia rutaecarpa*	OC-like cell model	Inhibited bone resorption and reduced the number of multinucleated cells	(82)
			MC3T3-E1 cell line	Promoted the p38-MAPK signaling	(83)
	Nomilin	*Citrus junos*	Mouse BMMs; Mouse RAW 264.7	Suppressed NFATc1 and MAPK signaling pathways	(84)
	Diosgenin	*Dioscorea nipponica*	OVX rats	Decreased the RANKL/OPG ratio	(85)
	Dioscin		Mouse BMMs cells; RAW264.7 cells; LPS- induced bone loss mouse	inhibiting the Akt signaling pathway	(86)
			MC3T3-E1 cells and MG-63 cells	Promoted osteoblasts proliferation and differentiation *via* Lrp5 and ER pathway	(87)
	Ophiopogonin D	*Ophiopogon japonicus*	OBs MC3T3-E1 cell; RAW264.7 cells; OVX mouse	Reducted oxidative stress *via* the FoxO3a-β-catenin signaling pathway	(88)
			Endothelium-specific Klf3 knockout mice	Inhibited Krüppel-like factor 3 (KLF3)	(89)
	Cycloastragenol	*Astragalus membranaceus*	MC3T3-E1 cells	Activated telomerase	(90)
	Hederagenin	*Hedera helix*	Mice BMMs; OVX mice	Inhibited RANKL-induced bone resorption and OC generation, activated MAPK signaling pathway (ERK and p38)	(91)
	Tubeimoside I	*Bolbostemma paniculatum*	Mice BMMs; RAW 264.7 cells; Male SD rats	Down-regulated NF-κB signaling pathway	(92)

### Monoterpenoids

Sweroside, an iridoid glycoside obtained from *Cornus officinalis* Sieb. et Zucc. (Shan Zhu Yu in Chinese), is commonly used in TCM for treating OP in postmenopausal women or elderly men (93). Emerging evidences demonstrated that sweroside increased the proliferation and suppressed the apoptosis of human MG-63 cells and rat OBs (17). Yan et al. observed that sweroside effectively promoted OB differentiation in bone marrow mesenchymal stem cells (BMSCs) through hyperactivating the mechanistic target of rapamycin complex 1 (mTORC1)/pS6 signaling pathway (19). Additionally, sweroside treatment induced the mineralization of bone matrix *via* modulating the expression of bone morphogenetic protein (BMP)-2/core binding factor alpha 1 (CBFA1)-mediated molecules in postmenopausal OP. Meanwhile, sweroside promoted the mineralization of MC3T3-E1 cells by activating p38 signaling pathway (16, 18). Swertiamarin, a structural analog of sweroside, is a secoiridoid glycoside extracted from *Enicostemma axillere* subsp. axillere (Gentianaceae) (94). It was evidenced that swertiamarin could promote OB differentiation and exhibit anti-inflammatory activity by regulating NF-κB/inhibitor of κB (IκB) and Janus kinase 2 (JAK2)/signal transducer and activator of transcription 3 (STAT3) signaling pathways. In addition, swertiamarin treatment markedly reduced RANKL/RANK expression and elevated osteoprotegerin (OPG) level, showing an excellent anti-osteoclastogenic activity (20–22).

*Morinda officinalis* HOW (Ba Ji Tian in Chinese) has been continuously used for more than 2,000 years in China as a tonic to nourish the kidney, strengthen bones, and enhance immune function in the treatment of OP (95, 96). It has been reported that the root extracts of *Morinda officinalis* showed therapeutic effect by suppressing bone resorption and enhancing bone formation on OP rat model induced by sciatic neurectomy and ovariectomy (97). He et al. observed that monotropein, a natural iridoid glycoside in the root extracts of *Morinda officinalis*, effectively attenuated lipopolysaccharide (LPS)- and ovariectomy-induced bone loss, and reduced inflammatory responses in MC3T3-E1 cells *via* inhibiting the activation of NF-κB (23). Furthermore, monotropein showed anti-osteoporotic effect by increasing bone mineral content (BMC), BMD, bone volume fraction (BVF), and decreasing the levels of interleukin (IL)-1, IL-6 and soluble RANKL in the serum of ovariectomized (OVX) mice (25). Meanwhile, monotropein treatment attenuated oxidative stress and increased the proliferation of OBs (24, 25).

Catalpol, the major bioactive iridoid glycoside isolated from *Rehmannia glutinosa* (Gaertn.) Libosch. ex Fisch. et C. A. Mey. Root (Dihuang in Chinese), is clinically used for OP treatment in China (6). Meng et al. showed that catapol suppressed RANKL-induced bone resorption in bone marrow-derived macrophages (BMMs) and RAW264.7 cells by reducing the ubiquitination of phosphatase and tensin homolog (PTEN), which subsequently inhibited the activations of NF-κB and protein kinase B (Akt) (26). Other reports also proved that catalpol treatment promoted the osteogenic ability of BMSCs and BMSC-dependent angiogenesis, partly *via* activation of JAK2/STAT3 axis and Wnt/β-catenin pathway (27, 28). Furthermore, Zhao et al. observed that catalpol could protect diabetic OP induced by high glucose treatment in MC3T3-E1 cells through regulating the migration and differentiation of OBs (29).

As a water-soluble monoterpene glucoside, paeoniflorin is the major bioactive components extracted from the root of *Paeonia lactifloral* Pall (98).. In antimycin A treated osteoblastic MC3T3-E1 cells, paeoniflorin attenuated cytotoxicity *via* improving the mitochondrial function. In addition, paeoniflorin also increased the differentiation of MC3T3-E1 cells and inhibited oxidative stress induced by methylglyoxal in the same cell model (30, 33, 98). In rats fed on high-carbohydrate/high-fat (HCHF) diet, paeoniflorin exhibited multiple pharmacological activities to prevent hyperlipidemia-induced OP. Intriguingly, paeoniflorin increased the trabecular and cortical parameters, as well as width and length of femur. Simultaneously, paeoniflorin rescued OB differentiation and the proliferation activities of bone turnover markers (99). Xu et al. reported that paeoniflorin suppressed bone destruction in collagen-induced arthritis (CIA) and decreased OC differentiation *in vitro* by down-regulating the activation of NF-κB (31). Wang et al. demonstrated that paeoniflorin suppressed OC generation and promoted OB formation *via* regulating NF-κB signaling pathway in BMMs and OVX mice (32).

Albiflorin, a monoterpene glycoside isolated from the roots of *Paeonia lactifloral* Pall., owns the ability to increase the differentiation of osteoblastic MC3T3-E1 cells (98). Kwang et al. found that albiflorin maintained mitochondrial function by reducing cytochrome c loss and cardiolipin peroxidation in MC3T3-E1 cells, which contributed to the inhibition of antimycin A-induced oxidative stress and toxicity (34). Another study showed that albiflorin treatment promoted the generation of OBs and expression of runt-related transcription factor 2 (RUNX2) through activating BMP-2/Smad and Wnt/β-catenin signaling pathways (35). Meanwhile, albiflorin up-regulated the levels of various osteogenic genes, such as osteocalcin (OCN), osteopontin (OPN), osteonectin (OSN), bone sialoprotein (BSP), and AP. In femur fracture rat model, albiflorin stimulated the expression of osteogenic genes in femoral tissue and promoted callus formation at the early stage during fracture recovery. Additionally, albiflorin could increase the expression of bone-related genes (35). This finding suggested that albiflorin motivated bone calcification, osteogenesis and bone formation, resulting in improving the fracture healing.

Bakuchiol is a prenylated phenolic monoterpene in the fruit of *Psoralea corylifolia* (L.) Medik (37, 100). And *Psoralea corylifolia* was used in TCM formulas to treat osteoporosis for a long history time (101). Recent researches indicated that *Psoralea corylifolia* and its major active ingredient bakuchiol possessed anti-OP activity (100, 102). Bakuchiol treatment significantly inhibited bone resorption and OC differentiation *via* the inhibition of Akt phosphorylation and c-jun nuclear translocation induced by macrophage colony stimulating factor (M-CSF) plus RANKL (38). In OVX Sprague-Dawley (SD) rats, bakuchiol treatment reduced bone loss through increasing Ca^2+^ and serum E2 concentrations, AP activity, and BMD, along with reduced inorganic P level (37). Li et al. found that bakuchiol significantly stimulated OB proliferation and differentiation (103). In addition, bakuchiol treatment prevented bone loss in OVX rats induced by estrogen deficiency and induced OB differentiation by up-regulating the Wnt signaling pathway (36).

Collectively, monoterpenoids can protect bone from erosion *via* targeting different signaling pathways. In OBs, catapol, albiflorin, and bakuchiol can activate Wnt/β-catenin signaling pathway; paeoniflorin and sweroside stimulate PI3K/Akt and MAPK signaling pathways respectively; swertiamarin inhibits RANKL/RANK signaling pathway; monotropein and swertiamarin suppress NF-κB signaling pathway. In OCs, catapol and paeoniflorin depress NF-κB signaling pathway; bakuchiol enhances PI3K/Akt signaling pathway.

### Sesquiterpenoids

Costunolide is sesquiterpene lactones derived from *Saussurea lappa* C.B. Clarke roots. A recent research showed that costunolide markedly induced bone mineralization and differentiation and increased cell growth, AP activity, and collagen synthesis in osteoblastic MC3T3-E1 cells *via* targeting diverse key proteins, such as estrogen receptor (ER), phosphoinositide 3-kinase (PI3K), extracellular signal-regulated kinase (ERK), protein kinase C (PKC), mitochondrial ATP-sensitive K^+^ channel, p38, and c-Jun N-terminal kinase (JNK) (39). Moreover, Cheon et al. observed that costunolide suppressed RANKL-induced OC differentiation *via* suppressing c-Fos transcriptional activity without affecting c-Fos expression (40).

*Saussurea lappa* C.B. Clarke has been used in clinic for decades as a TCM (104). Sesquiterpenes and sesquiterpene lactones are main bioactive constituent of this herb. As a member of sesquiterpene lactones, dehydrocostus lactone is extracted from the roots of *Saussurea lappa* and has been reported to exert various pharmacological activities including anti‐ulcer, anti‐tumor, anti‐inflammatory, and immunomodulation (42, 105). In mouse BMMs, dehydrocostus lactone attenuated the RANKL-dependent OC differentiation through modulating IκB kinase (IKK), JNK, nuclear factor of activated T cell cytoplasmic 1 (NFATc1), and nuclear factor-erythroid 2-related factor 2 (Nrf2). Moreover, it suppressed the activation of OCs through down-regulating the expression of integrin β_3_, PKC-β, and autophagy related 5 (43, 44). Besides, dehydrocostus lactone reduced RANKL‐induced OC formation and differentiation *via* modulating NF‐κB signaling pathway both *in vitro* and *in vivo* (41, 42).

Therefore, costunolide owns the ability to increase bone formation by modulating K_ATP_ channel and activating PI3K/Akt signaling pathway in OBs, and dehydrocostus lactone can decrease OC differentiation *via* inhibiting NF-κB signaling pathway.

### Diterpenoids

Euphorbia factor L1 (EFL1) is an active diterpenoid composition extracted from the seed oil of Chinese herb *Euphorbia lathyrism* L. (Qian Jin Zi in Chinese) (106). EFL1 inhibited RANKL-induced osteoclastogenesis by inhibiting c-Fos expression and NF-κB activation. Meanwhile, apoptosis induced by EFL1 in differentiated OCs resulted from caspase activation and enhanced Fas ligand expression. In mice, EFL1 ameliorated bone destruction induced by inflammation and ovariectomy. These findings demonstrated that EFL1 can block OC differentiation through modulating inflammatory responses and trigger Fas-regulated apoptosis, which offers the potential to treat OP caused by excessive Ocs (45).

Abietic acid is a bioactive diterpene isolated from *Pimenta racemosa* var. *grissea* which exhibits anti-obesity and anti-inflammatory activities (107). In RAW264.7 cells and mouse BMMs, abietic acid inhibited RANKL-induced OC formation *via* suppressing NF-κB and MAPK signaling pathways. It also decreased the expression of osteoclastic genes, such as NFATc1, tartrate-resistant acid phosphatase (TRAP), dendritic cell specific transmembrane protein (DC-STAMP), and c-Fos. In C57/BL6 male mice of osteolysis model induced by LPS, abietic acid significantly reduced the number of Ocs and the levels of inflammatory cytokines, including tumor necrosis factor (TNF)-α and IL-6 (46).

As a bicyclic diterpenoid lactone, andrographolide can be isolated from the leaves of traditional herb *Andrographis aniculate* (Burm. F.) Wall. Ex Nees in Wallich (Chuan Xin Lian). According to previous study, andrographolide has extensive pharmacological activities, such as anti-inflammation, anti-oxidation, anti-platelet aggregation, immunomodulation, and potential antineoplastic properties partly by targeting NF-κB (108–111). Andrographolide showed the capacity to protect breast cancer-induced bone loss (112) and inflammatory osteolysis (51, 113). Furthermore, andrographolide depressed osteoclastogenesis in BMMs by decreasing the expression of OC-related genes induced by RANKL and inhibiting bone loss and inflammation in OVX mice (48, 51). In addition, andrographolide promoted osteogenesis of mouse and rat BMSCs and blocked the inhibitory effect of TNF-α on OB formation and mineralization (47, 50). Other study indicated that andrographolide increased OPG expression and suppressed OC differentiation in MC3T3-E1 cells. It also stimulated the differentiation and survival of OBs, which increased bone deposition. Meanwhile, the study confirmed that andrographolide prevented bone loss and improved bone turnover rate in OVX rat model (49).

Carnosic acid, an abietane diterpenoid extracted from *Rosmarinus officinalis* (rosemary) and *Salvia officinalis* (common sage), displayed anti-angiogenic, anti-neoplastic, anti-oxidant and anti-HIV activities (114). Recent study had suggested the protective effect of rosemary against OP through effectively mitigated bone loss induced by calcium deficiency (115). Both in RAW 264.7 cells and mouse BMMs, carnosic acid decreased the osteoclastogenesis and reactive oxygen species (ROS) generation *via* activating Nrf2 and suppressing NF-κB and MAPK signaling pathways. The same results were also detected in C57BL/6 male mice of LPS-induced OP (52). Furthermore, Zheng et al. found that carnosic acid played a dual role *via* targeting sterol regulatory element-binding protein 2 (SREBP2) and estrogen-related receptor alpha (ERRα) to suppress RANKL-mediated osteoclastogenesis and restrained bone loss induced by ovariectomy (53).

Crocin, a diterpenoid glycoside carotenoid component of *Crocus sativus* L., shows various pharmacological activities (116, 117). It was observed that crocin treatment mitigated bone loss in metabolic syndrome-induced OP rat model (118). Meanwhile, this research showed anti-inflammatory and anti-oxidative activities of crocin which significantly decreased the production of IL-6, TNF-α, reduced glutathione (GSH), and superoxide dismutase (SOD). In RAW264.7 cells, crocin attenuated the dysfunction of OCs induced by methylglyoxal *via* modulating glyoxalase I, oxidative stress, and mitochondrial function (54). Moreover, Fatemeh et al. observed that crocin could effectively improve the differentiation of BMSCs, by inhibiting NF-κB signaling pathway activation, crocin treatment suppressed RANKL-induced bone resorption and OC formation (55, 119).

Kirenol is a bioactive diterpenoid compound derived from *Siegesbeckia orientalis* L. that was used as an anti-rheumatic TCM (120, 121). Kim et al. demonstrated that kirenol stimulated OB differentiation *via* activation of BMP and Wnt/β-catenin signaling pathways in MC3T3-E1 cells, which increased the levels of AP, OPN, type I collagen, and OB differentiation markers, as well as the OPG/RANKL ratio (57). Furthermore, kirenol treatment suppressed RANKL-induced OC formation and the NFATc1/Cav-1 signaling pathway in BMMs and OVX rats, consequently preventing ovariectomy-induced OP (56).

Tanshinone IIA is an abietane diterpenoid isolated from *Salvia miltiorrhiza* Bunge (Danshen) that is used for the treatment of trauma and fractures in clinical according to the dispelling stasis theory of TCM (122). 36 clinical trials used *Salvia miltiorrhiza* to treat different kinds of osteoporosis displayed high efficacy and low toxicity (123). Modern pharmacological studies showed that the ethanol extract of *Salvia miltiorrhiza* could inhibit trabecular bone loss by restraining bone resorption both in OVX and naturally menopaused mice (124). Tanshinone IIA blocked dexamethasone induced OB apoptosis through the suppression on NADPH oxidase (Nox) 4-derived ROS production. In addition, it blocked RANKL-mediated OC differentiation by decreasing the expression of c-Fos and NFATc1 (60). Tanshinone IIA could attenuate the formation of OCs by depressing the NF-κB, PI3K/Akt, and MAPK signaling pathways in OVX mice model (59). Zhu et al. found that tanshinone IIA administration prevented the harmfulness of oxidative stress and promoted the activity and functions of OBs in genetic OP model, Wnt1^sw/sw^ mice, through regulating the NF−κB signaling pathway (125). Recently, in streptozotocin (STZ)-induced C57BL/6 diabetic mice, tanshinone IIA treatment restrained the activity of renin that resulted in protecting OP (58). As another abietane diterpenoid constituent obtained from *Salvia miltiorrhiza*, tanshinone VI significantly suppressed the differentiation of OCs and bone resorption *via* down-regulating the expression of RANKL and activation of NF−κB (61).

Triptolide, the major active diterpenoid component isolated from *Tripterygium wilfordii* Hook F, has been used in TCM for hundreds of years to treat cancer and bone loss (126, 127). A recent study suggested that triptolide effectively suppressed the activation of NF-κB induced by RANKL, as well as tumor cell- and RANKL-induced OC formation (63). Triptolide showed the protective effects on bone loss both in old male rats and OVX C57BL/6 mice (62, 64). Triptolide could suppress RANKL-induced OC formation and prevented the bone resorption of OCs in BMSCs and RAW264.7 cells, resulting from inhibiting PI3K/Akt/NFATc1 signaling pathway.

Oridonin is an ent-kaurane diterpenoid extracted from the TCM herb *Rabdosia rubescens* (Hemsl.) Hara (128). As a plant metabolite, oridonin acts as an anti-tumor agent, angiogenesis inhibitor, apoptosis inducer, anti-asthmatic agent, and anti-bacterial agent (129, 130). Recent studies demonstrated that oridonin could maintain bone homeostasis (65, 66). In ovariectomy-induced OP mouse model, oridonin could protect bone loss *via* inhibiting osteoclastogenesis and enhancing osteogenesis by inhibiting interferon-related development regulator 1 (Ifrd1) and IκBα-mediated p65 nuclear translocation. Simultaneously, *in vitro* study revealed that oridonin motivated osteogenesis by Wnt/β-catenin signaling pathway and suppressed RANKL-induced OC formation in BMSCs.

In conclusion, diterpenoids are mostly investigated terpenoids that exert superior anti-OP efficacy by affecting various signaling pathways. In OBs, andrographolide, kirenol, and oridonim activate Wnt/β-catenin signaling pathway; andrographolide inhibits RANKL/RANK and NF-κB signaling pathways; tanshinone IIA blocks NF-κB signaling pathway. In OCs, euphorbia factor L1, abietic acid, carnosic acid, crocin, tanshinone IIA, and triptolide depress NF-κB signaling pathway; crocin, tanshinone IIA, and triptolide activate PI3K/Akt signaling pathway; andrographolide inhibits RANKL/RANK signaling pathway; abietic acid, carnosic acid, and tanshinone IIA inhibit MAPK signaling pathway; kirenol, tanshinone IIA, and triptolide depress NFATc1 signaling pathway; euphorbia factor L1 and carnosic acid promote Nrf2 signaling pathway.

### Triterpenoids

Lupeol is a major active lupine-type pentacyclic triterpenoid of *Sorbus commixta* Hedlund and *Celastrus orbiculatus* Thunb (131). Recently, lupeol has attracted the attention of researchers for its osteogenic activity. On one hand, lupeol significantly suppressed OC differentiation and bone resorption mediated by 1α, 25-(OH)_2_D_3_ and prostaglandin E2 (PGE2) *via* inhibiting the activities of MAPK and transcription factors (NF-κB, NFATc1, and c-Fos). On another hand, lupeol decreased hypercalcemic mediated bone loss in C57BL/6 mice (67). In addition, lupeol in *bombax ceiba* contributed to relieve bone fragility and fracture (132).

*Alismatis Rhizoma* is a famous traditional Chinese herb, which has been used for hepatoprotective, diuretic, hypolipidemic, anti-tumor, anti-inflammatory and anti-diabetic treatments for more than ten centuries (133, 134). More and more researches reported that the terpenoids constituents of this herb, such as the protostane triterpenes compounds Alisol B (69), Alisol A 24-acetate (71, 135), Alisol B 23-acetate (68), and Alisol C 23-acetate (70), own the protective activity against bone loss. Alisol A 24-acetate suppressed OC differentiation mediated by RANKL through downregulating NFATc1 and restraining the DC-STAMP and cathepsin K expression in mouse BMMs (71). Moreover, in OVX mice, alisol A 24-acetate and alisol C 23-acetate could effectively protect bone loss (70, 135). Alisol B suppressed the RANKL-induced osteoclastogenesis in mouse BMMs and stopped bone loss in 2-methylene-19-nor-(20S)-1a,25(OH)_2_D_3_ (2MD)-induced hypercalcemia mouse model (69).

As a member of the pentacyclic triterpenoids, oleanolic acid is a free acid or triterpenoid saponins in many Chinese herbs, such as Nvzhenzi (*Ligustri lucidi* W. T. Aiton), Baihuasheshecao (*Hedyotis diffusa*), Renshen (*Panax ginseng C. A. Meyer*), and Sanqi (*Panax Notoginseng (Burk.) F.H.Chen*). Nvzhenzi has been clinically applied in the treatment of OP for over 1,000 years (136). Chen et al. summarized more than 150 articles and reviews on the anti-osteoporosis activity of *Ligustri lucidi*. In TCM, *Ligustri lucidi* is believed to have anti-osteoporosis effects, improve liver and kidney deficiency and reduce lower back pain. Pharmacological experiments showed *Ligustri lucidi* improved bone metabolism and bone quality in OVX, growing, aged and diabetic rats *via* regulating PTH/FGF-23/1,25-(OH)2D3/CaSR, Nox4/ROS/NF-κB, and OPG/RANKL/cathepsin K signaling pathways (137) Oleanolic acid could suppress RANKL-mediated osteoclastogenesis in BMMs, and attenuate bone loss through decreasing the quantity of OC in C57BL/6 OVX mouse model (72). Furthermore, it has been proved that oleanolic acid modulated the ER alpha/miR-503/RANK signaling pathway to inhibit RANKL-induced osteoclastogenesis in RAW264.7 cells (138). In aged female rats and mature OVX mice, oleanolic acid regulated vitamin D metabolism to exhibit osteoprotective effect (73). The investigation with high-throughput metabolomics showed that oleanolic acid ameliorated the disordered metabolism state in glucocorticoid-induced OP rats (139). In addition, five oleanolic acid glycosides of *Achyranthes bidentata* also exerted inhibitory effect on the formation of OC-like multinucleated cells (OCLs) induced by 1α, 25-(OH)_2_D_3_ (140).

Ursolic acid, as the isomer of oleanolic acid, is a ubiquitous active triterpenoids constituent in traditional Chinese medicinal herbs, such as *Salvia miltiorrhiza* (141, 142), *Fructus ligustri lucidi* (143), and *Eriobotrya japonica* (144, 145). Ursolic acid exhibited multiple pharmacological activities, including anti-cancer, anti-inflammation, anti-anaphylaxis, and anti-aging (146–148). In recent years, ursolic acid has attracted the attention of researchers for its osteogenic activity. Lee et al. proved that ursolic acid induced the expression of OB-specific genes by activating NF-κB, MAPK, and activator protein-1. Moreover, they demonstrated the osteogenic activity of ursolic acid in a mouse calvarial bone model (74). As the two most abundant ingredients in *Fructus ligustri lucidi*, both ursolic acid and oleanolic acid regulated the expression of bone turnover markers and calcium balance in mature OVX rats. In addition, the combination of these two compounds significantly improved bone properties and vitamin D metabolism in aged female rats (143, 149). Tan et al. observed that ursolic acid prevents OC differentiation induced by RANKL in RAW 264.7 cells through targeting XPO5 (150).

Glycyrrhizic acid, as well as glycyrrhetinic acid, are extracted from the root of *Glycyrrhiza glabra* L., and glycyrrhizic acid is formed by the combination of pentacyclic triterpenoid glycoside and glycyrrhetinic acid (151). Both of them showed protective effects on glucocorticoid-induced OP (152). Glycyrrhizic acid and glycyrrhetinic acid could act as the ligands for glucocorticoid receptor (GR), which further modulated glucocorticoid resistance and ameliorated inflammatory responses by disrupting the GR-heat shock protein 90 (HSP90) (76, 153). Glycyrrhizic acid prevented glucocorticoid-induced OP in male SD rats through inhibiting the 11β-hydroxysteroid dehydrogenase type 1 enzyme (11β-HSD1) (75). Furthermore, Yamada et al. found that in an aging mouse model of periprosthetic osteolysis, glycyrrhizic acid alleviated inflammatory bone loss and increased senescence-protective sirtuins expression (77). In OVX mice model, glycyrrhizic acid treatment improved bone metabolism and suppressed OC differentiation *via* modulating NF-κB, ERK, and JNK signaling pathways (7, 154). Glycyrrhetinic acid inhibited osteoclastogenesis *via* decreasing RANKL-mediated association of RANK and TNF receptor associated factor 6 (TRAF6), and consequently inactivating the NF-κB and MAPK signaling pathways *in vitro* (BMMs and RAW264.7 cells) and *in vivo* (OVX C57BL/6 mice) (78).

Betulinic acid is a pentacyclic lupane-type triterpene derivative of *Betula pubescens* Ehrh., exhibiting multiple biological effects including osteogenic activity. Betulinic acid could enhance the proliferation, differentiation, and mineralization of osteoblastic MC3T3-E1 through regulating the BMP/Smad/Runx2 and β-catenin signal pathways (81). Furthermore, betulinic acid reduced RANKL-associated osteoclastogenesis *via* suppressing the MAPK and NFATc1 signaling pathways in BMMs isolated from C57BL/6 mice. In the osteoporotic C57/BL6 mice, betulinic acid prevented ovariectomy-induced bone loss (80).

Ginsenoside Rg1, a tetracyclic triterpenoid, is an active compound in *Panax ginseng* C. A. Meyer and *Panax japonicus* (T. Nees) C. A. Meyer, which acts as a neuroprotective agent and pro-angiogenic agent. Ginsenoside Rg1 promoted the proliferation and odontogenic/osteogenic differentiation of human dental pulp stem cells (hDPSCs), stimulated the proliferation of BMSCs, and suppressed the maturation and differentiation of OCs (79). Zishen Jiangtang Pill (ZJP) is a formula of Chinese medicine, which regulated bone metabolism in diabetic OP (DOP) and consequently exhibited a protective effect. As the primary active ingredient of ZJP, Ginsenoside Rg1 improved the ultrastructure and histomorphology of bone and islets in DOP rats (155).

Limonin is a tetracyclic triterpenoid of various TCM and fruits, such as *Evodia rutaecarpa*, *Coptidis rhizoma*, *Cortex chinensis phellodendri*, *bergamot*, *Aurantii fructus immaturus*, *Citri reticulatae pericarpium*, and citrus fruits (156). Early study showed that limonin significantly inhibited bone resorption and reduced the number of multinucleated cells with TRAP-positive nature in OC-like cell model (82). Otherwise, limonin treatment modulated the ERK and p38-MAPK signaling in osteoblastic MC3T3-E1 cell line to induce osteogenic differentiation (83).

Nomilin, a furan-containing triterpenoid isolated from medicinal citrus, showed inhibitory effects on RANKL-stimulated OC differentiation and bone resorption in RAW 246.7 cells and mouse BMMs cells, resulting from the inhibition of-NFATc1 and MAPK signaling pathways (84).

Diosgenin and dioscin are steroid sapogenin triterpenoids, which are extracted from *Dioscorea nipponica* Makino (157).. It was reported that diosgenin could suppress osteoclastogenesis and bone resorption. Meanwhile, it enhanced the osteogenic activity of OBs that contributed to increased bone formation *in vitro*, and anti-osteoporotic effect *in vivo* (85, 158–162). Diosgenin ameliorated bone loss by decreasing the RANKL/OPG ratio in OVX rats (85, 163) and retinoic acid-induced OP rats (164). Similarly, dioscin enhanced osteoblastogenesis and inhibited osteoclastogenesis to prevent ovariectomy-induced bone loss (165). In addition, dioscin blocked OC differentiation and bone resorption *via* inhibiting the activation of Akt signaling pathway (86). In human and mouse OB-like cell lines, dioscin promoted the proliferation and differentiation of OBs *via* Lrp5 and ER pathway (87).

Ophiopogonin D is a saposins triterpenoid extracted from the TCM *Ophiopogon japonicus* (L. f.) Ker-Gawl. and has been applied in clinical use for a long time. Ophiopogonin D suppressed ROS generation to exert anti-OP effects *via* the FoxO3a/β-catenin signaling pathway in both RAW264.7 and MC3T3-E1 cells. In RAW264.7 cells, ophiopogonin D decreased the expression of Osteoclastic genes and the activity of CTX1 and TRAP, which are bone degradation markers in serum. In MC3T3-E1 cells, ophiopogonin D significantly promoted cell proliferation and increased the gene levels of some osteogenic markers (88). Furthermore, Yang et al. highlighted that ophiopogonin D owned the ability to inhibit Krüppel-like factor 3 (KLF3), resulting in increased abundance of vessels in the bone tissue for bone formation (89).

As a pentacyclic triterpenoid compound, cycloastragenol is the aglycone derivative of astragaloside IV isolated from the root of *Astragalus membranaceus* (Fisch.) Bunge, which is a TCM used for thousands of years (166). Recent study reported that cycloastragenol might be a candidate drug to treat glucocorticoid-induced OP (GIOP) through alleviating the inhibition of osteogenic differentiation induced by dexamethasone (90). Yu et al. also observed that cycloastragenol treatment could improve bone formation, protect bone microstructure from degradation, reduce OC number, and augment bone biomechanical properties in both bone loss models induced by aging and D-galactose. Furthermore, cycloastragenol promoted the differentiation, viability, and mineralization of osteoblastic MC3T3-E1 cells. Cycloastragenol could also alleviate bone loss through increasing osteoactivin expression (167).

Hederagenin is a pentacyclic triterpenoid sapogenin extracted from *Hedera helix* (common ivy). In BMM cell model, hederagenin depressed the formation and bone (hydroxyapatite) resorption of OC induced by RANKL. Mechanism study revealed that hederagenin reduced the production of intracellular reactive oxygen species (ROS) and the activation of MAPK signaling pathway (ERK and p38), causing decreased induction of c-Fos and NFATc1. Similar to the *in vitro* effects, hederagenin treatment significantly prevented bone loss in OVX mice *via* inhibiting RANKL-induced bone resorption and OC generation (91). Meanwhile, hederagenin 3-*O*-(2-*O*-acetyl)-*α*-L-arabinopyranoside remarkably elevated the protein levels of BSP and osteocalcin and augmented AP activity (168).

Tubeimoside I, isolated from the Chinese medicinal herb *Bolbostemma paniculatum* (Maxim) Franquet (Cucurbitaceae), is a natural pentacyclic triterpenoid, and traditionally used for the treatment of snake venoms and inflammation. Recently, it was reported that tubeimoside I could inhibit the formation and function of OCs, as well as type 2 diabetes-induced decrease of bone mass in SD rats, resulting from down-regulating IκBα degradation which subsequently suppressed NF-κB transcriptional activity (92).

In summary, triterpenoids are potential anti-OP candidates with multi-target characteristics. In OBs, betulinic acid can activate Wnt/β-catenin signaling pathway; ophiopogonin D stimulates FoxO3a/β-catenin signaling pathway; ursolic acid, limonin, diosgenin, and dioscin promote MAPK signaling pathway. In OCs, diosgenin and dioscin enhance PI3K/Akt signaling pathway; lupeol, glycyrrhetinic, and tubeimoside I inhibit NF-κB signaling pathway; oleanolic acid inhibits RANKL/RANK signaling pathway; lupeol, betulinic acid, nomilin, and hederagenin depress MAPK signaling pathway; lupeol, alisol B, betulinic acid, and nomilin block NFATc1 signaling pathway.

## Conclusion and Prospects

TCM has been widely used around the world for thousands of years to treat various diseases. These *in vivo* and *in vitro* findings discussed above demonstrate that terpenoids in natural Chinese medicine own the potential ability to provide therapeutic benefits for OP treatment.

Although terpenoids are beneficial for OP treatment, some terpenoids have been reported to be toxic. Cantharidin, a monoterpene obtained from *Mylabris phalerata* showed nephrotoxicity by suppressing the lactate dehydrogenase expression and intracellular release (169). Diterpene compound Pekinenin C and pekinenal also exhibited serious cytotoxicity intestinal toxicity (170). Thus, modification of their structures for lower toxicity and stronger efficacy are needed. For example, the quinoxaline derivative of oleanolic acid, QOA-8a, could not only inhibit bone resorption but also stimulate bone formation, playing dual roles in anti-OP (171). Meanwhile, the addition of quinoxaline contributed to lower cytotoxicity (172). Comparing with andrographolide itself, its derivative 14-deoxy-11,12-didehydroandrographolide showed stronger anti-osteoclastogenesis effect with significantly reduced cytotoxicity (173, 174). Therefore, structure modification will be an optional strategy for anti-OP drug development based on natural terpenoids. In addition, other problems, such as poor water solubility, short half-life, poor stability, and low bioavailability, severely limit the development and clinical use of TCM. The application of modern technologies (nanotechnology and co-crystallization) can overcome these short comings (175–177). Hence, for those terpenoids with perfect anti-OP efficacy but poor water solubility, we can apply nanoparticles in the drug delivery.

Nowadays, though a massive of studies reveal the anti-OP effects and molecular mechanisms of terpenoids, most of their direct targets as well as regulation mechanisms have not been illustrated. Several advanced technologies, such as proteomics (178) and systems pharmacology-based approaches (179, 180), have offered effective tools to identify potential targets of natural terpenoids. Proteomics and systems pharmacology-based approaches could perform the large-scale study of proteins and the major targets of most compounds. On the one hand, it is helpful to explain the exact pharmacological mechanism for pre-clinical drug development. On the other hand, the screening of terpenoids targeted proteins in OP treatment benefits researchers for understanding the pathogenesis of osteoporosis.

Moreover, TCM not only exerted anti-OP functions alone through diverse signaling pathways, but also showed enhancing effects *via* combining with clinically used hormones (estrogen or growth hormone) to prevent bone loss (181). This combination can avoid possible toxic side-effects and improve clinical efficacy (182). In the future, more in-depth and high-quality clinical researches are essential to ensure the safety, efficacy, and specificity of the terpenoids, which will provide more evidence for the candidates in efficiently anti-osteoporotic applications.

## Author Contributions

JF and YZ: conceptualization. YZ and ML: writing — original draft preparation. QJ, HK, QL, and L-FZ: editing, and revising. JF: supervision. All authors contributed to the article and approved the submitted version.

## Funding

The work was supported by the National Natural Science Foundation of China (No.82074278), the Guangdong Basic and Applied Basic Research Foundation (No. 2021A1515110584), Special Foundation of Guandong Educational Committe (No. 2021ZDZX2001), and Guangdong Province Science and Technology Plan International Cooperation Project (No. 2020A0505100052).

## Conflict of Interest

The authors declare that the research was conducted in the absence of any commercial or financial relationships that could be construed as a potential conflict of interest.

## Publisher’s Note

All claims expressed in this article are solely those of the authors and do not necessarily represent those of their affiliated organizations, or those of the publisher, the editors and the reviewers. Any product that may be evaluated in this article, or claim that may be made by its manufacturer, is not guaranteed or endorsed by the publisher.
